# AI-enabled wearable cameras for assisting dietary assessment in African populations

**DOI:** 10.1038/s41746-024-01346-8

**Published:** 2024-12-05

**Authors:** Frank P.-W. Lo, Jianing Qiu, Modou L. Jobarteh, Yingnan Sun, Zeyu Wang, Shuo Jiang, Tom Baranowski, Alex K. Anderson, Megan A. McCrory, Edward Sazonov, Wenyan Jia, Mingui Sun, Matilda Steiner-Asiedu, Gary Frost, Benny Lo

**Affiliations:** 1 The Hamlyn Centre for Robotic Surgery, Imperial College London, London, UK; 2 Department of Population Health, London School of Hygiene & Tropical Medicine, London, UK; 3 College of Electronics and Information Engineering, Tongji University, Shanghai, China; 4 Department of Pediatrics, USDA/ARS Children’s Nutrition Research Center, Baylor College of Medicine, Houston, TX USA; 5 Department of Nutritional Sciences, University of Georgia, Athens, GA USA; 6 Department of Health Sciences, Boston University, Boston, MA USA; 7 Department of Electrical and Computer Engineering, The University of Alabama, Tuscaloosa, AL USA; 8 Department of Electrical and Computer Engineering, University of Pittsburgh, Pittsburgh, PA USA; 9 Department of Neurological Surgery, University of Pittsburgh, Pittsburgh, PA USA; 10 Department of Nutrition and Food Science, University of Ghana, Accra, Ghana; 11 Section of Nutrition, Department of Metabolism, Digestion and Reproduction, Imperial College London, London, UK

**Keywords:** Nutrition, Public health, Health care

## Abstract

We have developed a population-level method for dietary assessment using low-cost wearable cameras. Our approach, EgoDiet, employs an egocentric vision-based pipeline to learn portion sizes, addressing the shortcomings of traditional self-reported dietary methods. To evaluate the functionality of this method, field studies were conducted in London (Study A) and Ghana (Study B) among populations of Ghanaian and Kenyan origin. In Study A, EgoDiet’s estimations were contrasted with dietitians’ assessments, revealing a performance with a Mean Absolute Percentage Error (MAPE) of 31.9% for portion size estimation, compared to 40.1% for estimates made by dietitians. We further evaluated our approach in Study B, comparing its performance to the traditional 24-Hour Dietary Recall (24HR). Our approach demonstrated a MAPE of 28.0%, showing a reduction in error when contrasted with the 24HR, which exhibited a MAPE of 32.5%. This improvement highlights the potential of using passive camera technology to serve as an alternative to the traditional dietary assessment methods.

## Introduction

According to the 2016–2025 Nutrition Strategy of the World Health Organisation (WHO), the organisation collaborates with Member States to ensure universal access to healthy diets and effective interventions aimed at ending all forms of malnutrition. This goal is particularly important in African countries, which are undergoing a nutrition transition. Despite a significant proportion of the population still suffering from undernutrition in Africa, overweight is increasing in certain urban areas, along with the burden of diet-related diseases, contributing to the so-called double burden of malnutrition^1^. In order to plan policies to eradicate or reduce malnutrition, it is essential to have information on habitual food and nutrient intake at the individual and household levels. A commonly used tool in nutritional epidemiology studies is the 24-Hour Dietary Recall (24HR) in which participants recall and report the food types and portion sizes to experienced dietitians. However, this traditional self-reporting method is labour-intensive, expensive, and inevitably leads to a biased and inaccurate dietary analysis due to dependence on self-report. Moreover, the burdens associated with the requirement to self-report diet on multiple occasions can influence eating behaviour, study recruitment, and importantly, subsequent retention, thus biasing participant demographics. In recent years, efforts have been made to minimise reporting bias and make the process more efficient by using various dietary assessment tools, such as mobile phones to record dietary intake. However, these methods require access to mobile technology, computer literacy, and certain levels of human intervention, which may pose significant problems when deploying population-level dietary assessments in low-and middle-income countries (LMICs). To address this issue, our research group proposed a passive pipeline, EgoDiet, using simple and low-cost wearable cameras to capture food-eating episodes continuously and automatically, which can minimise the burden and technical requirements for users in dietary information collection and analysis. Using low-cost wearable cameras, this approach can potentially address the challenges of deploying population-level dietary assessments in LMICs and improve the accuracy of dietary analysis. Apart from this, due to its purely passive nature, EgoDiet not only enables data collection and analysis with minimal human involvement, but it also has clinical applications. For instance, patients with chronic diseases such as diabetes, cardiovascular diseases, or kidney diseases require strict dietary monitoring. The system can provide healthcare providers with more precise data on patients’ food intake, allowing for more tailored and effective dietary recommendations and interventions. For users suffering from malnutrition, the system can help in closely monitoring their food intake to ensure they are receiving the required nutrients. This real-time monitoring can assist in adjusting dietary plans promptly based on the patient’s actual consumption. In broader public health initiatives, the passive camera technology also moves the understanding of food intake closer to the ground truth of the nutritional intake of family members, providing insight into how food is distributed within family groups. Additionally, it offers a more accurate picture of the extent of malnutrition and the specific nutrients that are deficient in particular areas. These findings have the potential to facilitate the implementation of effective nutrition actions and policies in close collaboration with local governments.

EgoDiet is a comprehensive dietary assessment pipeline that consists of various modules. The EgoDiet:SegNet module is a network that utilises a Mask Region-based Convolutional Neural Network (Mask R-CNN) backbone^2^ and the network is optimised for the segmentation of food items and containers in African cuisine. This enables the recognition of food items, tracking and detection of containers at multiple scales. Furthermore, it enables the extraction of useful portion size-related features for further dietary analysis. The EgoDiet:3DNet module is a depth estimation network with encoder-decoder architecture^3^ which estimates the camera-to-container distance, as well as reconstructs the three-dimensional (3D) models of the containers. This allows for the rough determination of container scale without the need for costly depth-sensing cameras. The EgoDiet:Feature module is a feature extractor that extracts important portion size-related features from the segmentation masks generated by the EgoDiet:SegNet module and the 3D models obtained from the EgoDiet:3DNet module. Algorithms are developed to utilise these features in order to deduce information for dietary analysis, such as the Food Region Ratio (FRR), which indicates the proportion of the region occupied by each food item in a container. In order to design a system that is compatible with wearable cameras worn at different body positions, the orientation of the cameras is taken into account and a new indicator, Plate Aspect Ratio (PAR), is introduced. PAR indicates the height-width ratio of the container and enables the rough estimation of the tilting angle of the cameras in a simple and efficient manner. This particular problem has seldom been discussed before since active methods (e.g. such as those relying on the users to use their mobile phones to take photos of the food items) can select their own capturing angles and distance which suit their requirements, by contrast to our approach that only relies on a passive camera without any constraints on capturing positions. EgoDiet:PortionNet is the last module which estimates the portion size (i.e. in weight) of the food consumed. This task is particularly challenging since there are insufficient representative training data to train a portion size estimation model due to labour intensive and complicated annotation procedure, which either requires a standardised weighting scale to measure the weight for each particular food item or uses water displacement method to measure the volume. Consequently, implicit feature extraction using deep neural networks becomes difficult and inefficient (i.e. considered as a few shot regression problem). Instead of training the portion size estimation models that use large amounts of labelled data, we target and make good use of those task-relevant features extracted from EgoDiet:Feature with relatively little labelling to train our models. Similar idea has also been explored in a recent research work^4^.

To accurately quantify an individual’s food intake, measuring the actual amount of foods consumed (i.e. portion size) is critical for dietary assessment. With the recent development in computer vision techniques, different vision-based methods have been proposed to solve the problem of portion size estimation^5^. Specifically, the methods can be divided into several main categories ranging from stereo-based^6,7^, model-based^8–10^ and perspective transformation^11–13^ approaches. Although these methods show promising results in portion size estimation, they are mostly designed for methods which rely on active capturing of food intake (e.g. using a mobile phone app to capture the food images^14–17^), and thus difficult to implement in studies with passive capturing wearable cameras (where the wearable cameras continuously capture the food images automatically). For instance, stereo-based method has several constraints on the capturing angles, e.g. at least two images should be taken from different viewing angles^13^, to achieve feature matching and 3D reconstruction which in turn makes this method difficult to be used along with wearable cameras worn by the users. Another concern is that stereo-based approach relies heavily on feature matching between frames in order to obtain the 3D geometry of the food items. However, under low lighting conditions in LMIC households, the food surface often do not appear to have distinctive texture and characteristics which makes the stereo matching not possible. For model-based approach, it calculates the food volume by matching the food items in the image with the pre-built 3D food models. For instance, a research group proposed a virtual reality approach by superimposing 3D models with known volume onto the images with the food items^18^. However, this method requires certain human intervention, such as rotating and scaling the 3D models to match the food items on the image, which makes the method only suitable for use in active capture of food intake. Most importantly, the major concern is that model-based approach only measures initial portion size (i.e. the quantity of foods at the start of eating). It does not provide an estimation of the consumed portion size (i.e. the total quantity of foods eaten), contributing to large errors in nutrient intake assessments. Similar to stereo-based approach, perspective transformation approach also requires users to use hand-held cameras and take images from specific locations, e.g. putting the mobile phones on the table^11^, capturing images using bird-eye view^19^. Detailed information of various recent works based on active approach can be found in Table 1. Compared to previous works, our proposed passive method has fewer limitations, making it more versatile and convenient in providing portion size estimation. Beyond offering convenience, passive systems record important dietary behaviours such as the eating priority, personal food preferences, and meal timings. The sequence of food consumption reflects an individual’s prioritisation of different food items, a factor that can significantly differ due to cultural and personal variations. Food preference plays a crucial role in dietary assessment as it influences food choices and consumption patterns. By considering eating priority and food preferences, dietary assessments can be more accurate and personalised. Furthermore, the timing of meals holds considerable importance, influencing metabolic processes and overall health. Monitoring meal times aids in pinpointing irregular eating patterns or habits that could alter nutritional intake and affect one’s well-being. Therefore, we believe passive methods represent a new and innovative way for dietary assessment (See Supplementary Table 1 for more recent works using passive methods).

**Table 1:** Descriptions of recent research works utilising active methods in portion size estimation^a^

Recent works	Methods	Descriptions	Discussions	Error
Rahman et al.^7^	Utilise a two-view stereo 3D reconstruction method (Stereo-based method)	Capable of performing 3D reconstruction on irregularly shaped food items, such as fried rice or noodles	May encounter difficulties with objects that have less texture, such as basic ingredients like eggs and oranges	MAPE: 7.7% (in volume)
Dehais et al.^6^	Utilise a two-view stereo 3D reconstruction method (Stereo-based method)	An enhanced RANSAC method, faster than traditional dense reconstruction techniques	Two angles may not adequately capture the full structure of food items, especially their rear aspects	MAPE: 8.2%–9.8% (in volume)
Gao et al.^42^	Utilise a SLAM-based 3D reconstruction method (Stereo-based method)	With point cloud completion algorithm to overcome limited viewing angles	Requiring a strong assumption, it cannot estimate asymmetrical food items	MAPE: 16.4%–27.9% (in volume)
Xu et al.^10^	Utilise 3D-model generation and pose estimation (Model-based method)	Precisely accurate estimation using pre-built models	Handling new and unseen shapes can be challenging, a library of 3D models is required for this approach	MAPE: 3.6%–12.3% (in volume)
Sun et al.^18^	Utilise a virtual reality method (Model-based method)	A robust and easy-to-use system for estimating volume	Model-based methods often fail to predict leftover food	RMSE: 20.5% (in volume)
Fang et al.^8^	Utilise a single-view reconstruction method based on both shape templates and prism models (Model-based method)	Automatically estimating volume using geometric contextual information without the need for manual initialisation of estimation parameters	Assuming that the height of the entire horizontal cross-section is constant in using prism model method	Error in energy < 6.0%
Fang et al.^43^	Utilise the depth camera to estimate volume (Depth camera-based method)	With a depth sensing device, there is no need for a reference object with known size	Mobile devices do not always come equipped with a depth camera; This method could lead to an overestimation problem	MAPE: 11.0%–33.9% (in volume)
Jia et al.^12^	Utilise the plate method and LED method (Perspective transformation method)	This method is semi-automatic and employs a plate with pre-measured dimensions as a reference object	Limitations exist on the application of this method in studies involving a large number of subjects	MAPE < 10% (wireframe fitting method) (in volume)
Pouladzadeh et al.^44^	Utilise an area measurement technique to conduct volume estimation (Perspective transformation method)	Capable of handling irregularly shaped foods, and this system utilises the user’s thumb as the reference object	Strict constraints on image capture angles, requiring images to be captured from both above and the side	Maximum error in volume < 10.5%
Yang et al.^11^	Utilise an unique image capture technique for determining the actual scale of food items (Perspective transformation method)	Estimation of food volume without the requirement of a fiducial marker	It is difficult to capture an image of the food with the smartphone’s bottom placed on the tabletop	MAPE: 16.7% for large food objects (in volume)
Meyers et al.^17^	Utilise a depth estimation network to facilitate 3D reconstruction of food items (Deep learning method)	A large dataset of paired RGB and depth images is collected to train the depth estimation network	The method exhibits significant errors in both depth map and volume predictions, while its practical application is still constrained to laboratory settings	Mean volume estimation error: 50-400 ml
Christ et al.^23^	Utilise FCNN to predict the depth map and estimate the Bread Units (BUs) via Resnet-50 (Deep learning method)	Validate the possibility of utilising neural networks for the estimation of depth and BUs	The network cannot be trained end-to-end and exhibits a high error rate in predicting BUs	RMSE: 1.5 bread units
Chokr et al.^45^	Utilise a machine-learning-based approach to predict the amount of calories from food images (Deep learning method)	Demonstrate the effectiveness of ML across various dietary assessment tasks, ranging from the food recognition to portion size estimation and ultimately to the precise calorie prediction	The current system is capable of processing only one individual food item at a time. An extension is required to handle meals comprising various foods presented simultaneously	A reduction of over 93% in MAE compared to a baseline approach (in energy)
Fang et al.^46^	Utilise a generative adversarial network for estimating the distribution of food energy (Deep learning method)	Mapping of the food images to the food energy distribution through an end-to-end network	Obtaining paired RGB images and their corresponding energy distribution is a challenging task	MAPE: 10.9% (in energy)
Lo et al.^5^;	Utilise an AI-based 3D reconstruction method to conduct volume estimation (Deep learning method)	Address visual occlusion problem with AI-based point completion method for more accurate volume estimation	Obtaining paired sets of partial and complete point clouds is a non-trivial task	MAPE: 15.3% (in volume)
Pfisterer et al.^47^	Utilise a RGB-D camera and a deep convolutional Encoder-Decoder Food Network with Depth-refinement (EDFN-D) (Deep learning method)	Capable of estimating the volume of remaining food relative to reference portions in whole and modified texture foods	May struggle to estimate volume when the depth information is not available and food in a diverse range of containers	Mean percent intake error: 4.2%

^a^It should be emphasised that the previous studies on estimating portion sizes have solely relied on self-made test datasets that only cover a small selection of food items. As a result, there is no benchmark that enables researchers to conduct a comprehensive comparison with previous methods in terms of accuracy. Therefore, it is advisable to assess portion size estimation techniques based on their practicality and feasibility and to determine if they overcome the aforementioned limitations. A more comprehensive description and limitations of research works using active methods in portion size estimation can be found in our previous work^13^. Recent works based on passive monitoring can be found in Supplementary Table 1. MAPE, MAE, and RMSE refer to Mean Absolute Percentage Error, Mean Absolute Error, and Root Mean Square Error respectively.

## Results

### Study areas and data collection protocol

**Fig. 1:**
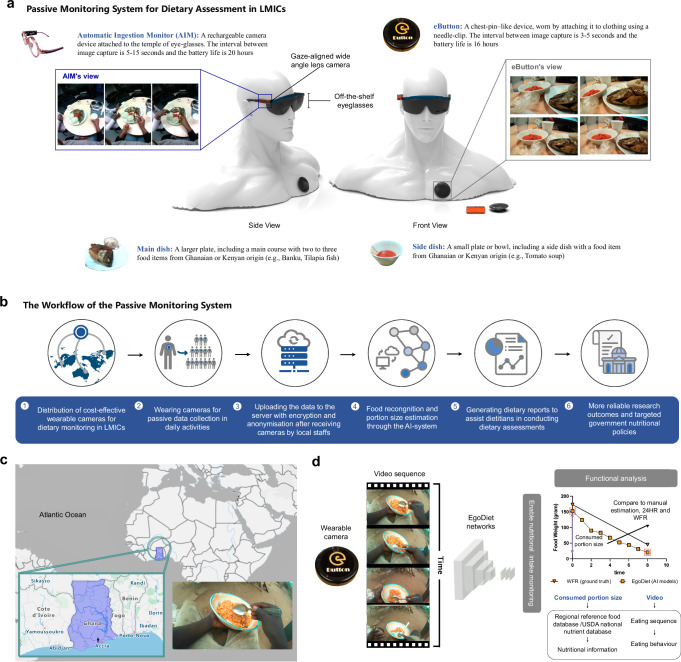
An illustration diagram of the AI-enabled passive monitoring system for dietary assessment. **a** The prototype of the customised wearable cameras (AIM^20^ and eButton^21^ devices). The descriptions of the wearable cameras are shown. **b** The workflow of the passive monitoring system for dietary assessment. The wearable cameras were deployed, set up prior to the study and recollected after the study by our staff in the field site. The collected data was uploaded to the cloud server and fed into the trained AI models for recognising the food categories and estimating consumed portion size. The system can generate a report showing the consumed food items and their corresponding portion size for each individual/ household in a particular region to assist dietitians in conducting nutrition surveys. **c** The field studies were conducted in both London (Study A) and Ghana (Study B) with populations of Ghanaian and Kenyan origin. **d** Passive monitoring enables the capturing of eating sequences, the identification of food items left unconsumed by the participants, and the record of meal timing and duration, from which the habits and eating behaviours of the participants could be observed. In order to demonstrate the efficacy of EgoDiet compared to traditional methods in dietary assessment, we compared our method against two commonly used dietary assessment techniques. In Study A, we compared EgoDiet to manual visual-based portion size estimation carried out by experienced dietitians, while in Study B, we compared EgoDiet to 24HR. Weighed Food Records (WFRs) were used as the ground truth in both studies. The two boxed spots (in red) are marked to indicate the two-time frames used to calculate consumed portion size. Once we know the consumed portion size, we can calculate the nutritional information using regional or national nutritional databases.

The study areas were located in both London (Study A) and Ghana (Study B). In Study A, our research group conducted a feasibility study to evaluate the functionality of two customised wearable cameras namely the Automatic Ingestion Monitor (AIM)^20^ and eButton^21^ (Fig. 1a), and the performance of EgoDiet on dietary analysis for sub-Saharan African populations living in London. The AIM is a gaze-aligned wide angle lens camera which can be attached to the temple of eyeglasses (eye-level), while the eButton is a chest-pin-like camera which can be worn using a needle-clip (chest-level). Captured images on both cameras are stored on SD cards, with the capacity to store ≤ 3 weeks of data. To conduct the feasibility study, real-life video footages with the scenarios of users eating food items of Ghanaian and Kenyan origin were captured to be used as a test dataset. 13 healthy subjects of Ghanaian or Kenyan origin, aged ≥18, were recruited to participate in the study held at the National Institute for Health Research Clinical Research Facility (CRF). Before each study started, a standardised weighing scale (i.e. Salter Brecknell) was used to first pre-weigh the food items. Preweighed food items were placed on the food containers and presented to the subjects, and the wearable cameras were worn by the participants during eating. Instead of finishing all the food items, the subjects were asked to just eat until full. Then the food remnants were post-weighed and recorded, so that the difference between the pre-weighed and the post-weighed food items can be measured as the ground truth of the consumed food weight (i.e. This method is also known as Weighed Food Records (WFRs), which provide detailed quantitative information on an individual’s diet, such as consumed portion size. WFRs are considered a gold standard method of dietary assessment and are often used to validate other dietary assessment methods, such as Food Frequency Questionnaires and 24HR). The collected raw video footages were uploaded to the cloud server and fed into the trained AI models (EgoDiet) for predicting the consumed food categories and portion size. In Study B, our research group further conducted a study to evaluate the performance of our passive cameras, estimating dietary intake of populations in Ghana under free living conditions (Fig. 1c). A total of 10 households in Ghana were recruited, typically consisting of adults (mother and father) and adolescents. All participants had provided written consent to participate in the study. Our field staff visited the targeted households in the morning and dispatched the wearable cameras. The field staffs stayed at the household only for a certain of time (i.e. around 30 min) to ensure the devices were properly worn. In the evening, our staff would return to the households after the final household meal was eaten to gather all the devices. Similar procedures were conducted that we uploaded the anonymised and encrypted footage to the cloud server for dietary assessment (Fig. 1b). In order to demonstrate the efficacy of EgoDiet against traditional methods in dietary assessment, experienced dietitians/nutritionists were also involved in our study to perform portion size estimation (i.e. estimate the food consumption of the participants manually based on the camera images captured), and their estimation was then compared against the output of our passive method. The team included three professionals from the USA, Ghana, and the UK, each with over 20 years of experience in dietary assessment methodologies. Additionally, two dietitians from Ghana also contributed to ensuring reliable portion size estimations. To ensure the fairness of the comparison, the accessors were trained to be well familiar with the foods of Ghanaian and Kenyan origin and the technique of portion size estimation (Fig. 1d). Note that a detailed study protocol has been delineated in our prior publication^1^, which extensively describes the research objectives, the data collection process, and important considerations. Further discussions on the acceptability, functionality and validity of our system are elaborated in a subsequent analysis paper^22^. These publications collectively contribute to a comprehensive understanding of our system’s potential as an alternative to traditional dietary assessment methods.

### Design of the modules and validation

**Fig. 2:**
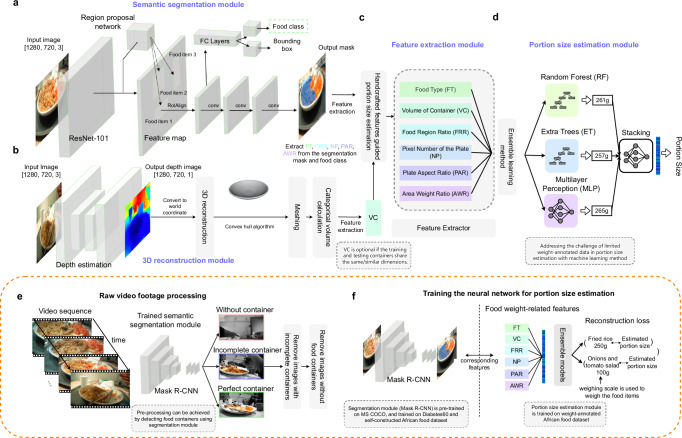
A detailed implementation of the EgoDiet pipeline. **a** Semantic segmentation module. It is implemented using the Mask R-CNN architecture, whose primary task is to segment African food items and food containers from passively captured images. Within this framework, 15 categories of African food items have been trained in the Mask R-CNN model. **b** 3D reconstruction module. It is a depth estimation network with encoder-decoder architecture, with the objective to infer depth maps from RGB images, thereby obtaining corresponding 3D models from which volume can be calculated. This volume is utilised as a rough estimation of scale and serves as a feature input for subsequent network processes. **c** Feature extraction module. This module extracts the following features from the pixels representing food and food containers within the segmentation masks: FT, FRR, PAR, NP, AWR. **d** Portion size estimation module. Utilising features extracted by the preceding network, this module trains a network capable of estimating food weight. An ensemble method comprising various machine learning techniques is employed. **e** Raw video footage processing through Mask-RCNN, a model structured similarly to what is depicted in semantic segmentation module. The focus is to accurately identify food containers and evaluate the completeness of the captured containers. **f** The training procedures of the EgoDiet. Our main network training can be divided into two parts: firstly, the semantic segmentation module, which is trained using annotated masks, and secondly, the portion size estimation module, which is trained using weight-annotated data.

Our pipeline performs segmentation (EgoDiet:SegNet) (Fig. 2a) followed by depth estimation and 3D reconstruction (EgoDiet:3DNet) (Fig. 2b), portion size-related features extraction (EgoDiet:Feature) (Fig. 2c) and, finally, portion size estimation (EgoDiet:PortionNet) (Fig. 2d) from the captured video frames. These four modules, trained/designed for different purposes, can be used individually or combined in different ways.

#### Segmentation module: EgoDiet:SegNet

Instead of simply using detection methods which only indicate the location of the food items and contain little information about the portion size, this module can generate the segmentation masks of food items and the containers. EgoDiet:SegNet is based on the Mask R-CNN architecture, and we optimised it for pixel-wise segmentation and integrated into the EgoDiet. We further applied transfer learning onto the weights of the Mask R-CNN backbone pre-trained on the Microsoft Common Objects in Context (MS COCO) dataset with 328k images. This boosts the performance of the African food items detection and segmentation. Afterwards, the module was trained using self-annotated frames from videos captured in the field site (See 'Methods') and the subset of diabetes60 dataset^23^ (Fig. 2f). The data collection methodology of the Diabetes60 dataset closely aligns with our passive monitoring approach, particularly in terms of the camera angles and distances employed during image capture. Furthermore, we applied image augmentation to increase the network robustness against invariances and therefore increase its generalisability. Note that image segmentation is an important aspect of the research; however, this study primarily focuses on the portion size estimation, and therefore, its coverage is relatively limited. It is also worth noting that image segmentation is quite mature, and there are numerous existing approaches and techniques available nowadays.

#### Segmentation performance on real-life video footage with eating scenarios

We first evaluated the performance of EgoDiet:SegNet on raw video footage captured in Study A (London) (i.e. include both images captured by eButton and AIM). After raw video footage processing (See 'Methods'), the frames were divided into three categories (i.e. with container, incomplete container and without container) (Fig. 2e). Note that the following validation study was conducted using the frames only with containers. To investigate the generalisability of the model in food segmentation, we present the qualitative results of the semantic segmentation applied on wearable cameras. As shown in the Supplementary Fig. 1, the network demonstrates that it has great potential in dealing with several challenging scenarios. With the semantic masks of the food items, the food categories can also be deduced as a sub-product. We evaluated both frame-level accuracy, wherein our model is applied to every frame to conduct food recognition, and scenario-level accuracy in food recognition, which averages the estimation of food categories for each scenario. Unlike food recognition using a single frame, averaging the results across the whole scenario could potentially improve the food recognition rate, showing the advantage of the passive method compared to most of the existing active approaches. Further improvements can also be achieved by annotating more images with food remnants (i.e. food items with small portion sizes) to train and refine the neural network.

#### Depth estimation and 3D reconstruction module: EgoDiet:3DNet

To facilitate large-scale studies and minimise the impact on the participants’ normal behaviour, a simple and low-cost wearable RGB camera is used to estimate portion size, rather than using the costly sensors with depth-sensing capability. This creates a new challenge due to scale ambiguity issues. To address this, we propose EgoDiet:3DNet, a neural network with encoder-decoder architecture pre-trained on the NYU Depth V2 dataset (i.e. a dataset with video sequences from a variety of household scenes captured by RGB and depth cameras), with relatively little labelling using task-relevant images. Similar ideas have been explored in previous publications^17,24^. The goal of this module is to obtain a rough scale for food containers, which facilitates the subsequent estimation of food portion size. To achieve this, we re-project the inferred depth images to the world coordinate system to obtain the 3D point cloud of the containers. We then apply the convex hull algorithm to the 3D point cloud to form a mesh, from which we can compute the volume of the food containers. The main advantage of using EgoDiet:3DNet to estimate container volume over previous approaches is that it takes the advantage of the coupled nature of depth and volume, and replaces the volume annotation with depth annotation, which can be easily obtained using stereo/depth cameras. We consider volume estimation as a downstream task of depth estimation and break the volume estimation task into two separate parts (i.e. depth estimation and 3D reconstruction), which simplifies the training process without requiring paired RGB images with ground truth volume of food containers. In contrast, existing approaches such as using Convolutional Neural Networks (CNN) to regress container volume require large amounts of volume-annotated data to learn contextual information from the environment. Such approach is very expensive and impractical for large-scale dietary assessment studies in the wild.

#### EgoDiet:3DNet demonstrates potential for comparable performance to depth sensors in food container volume estimation

**Fig. 3:**
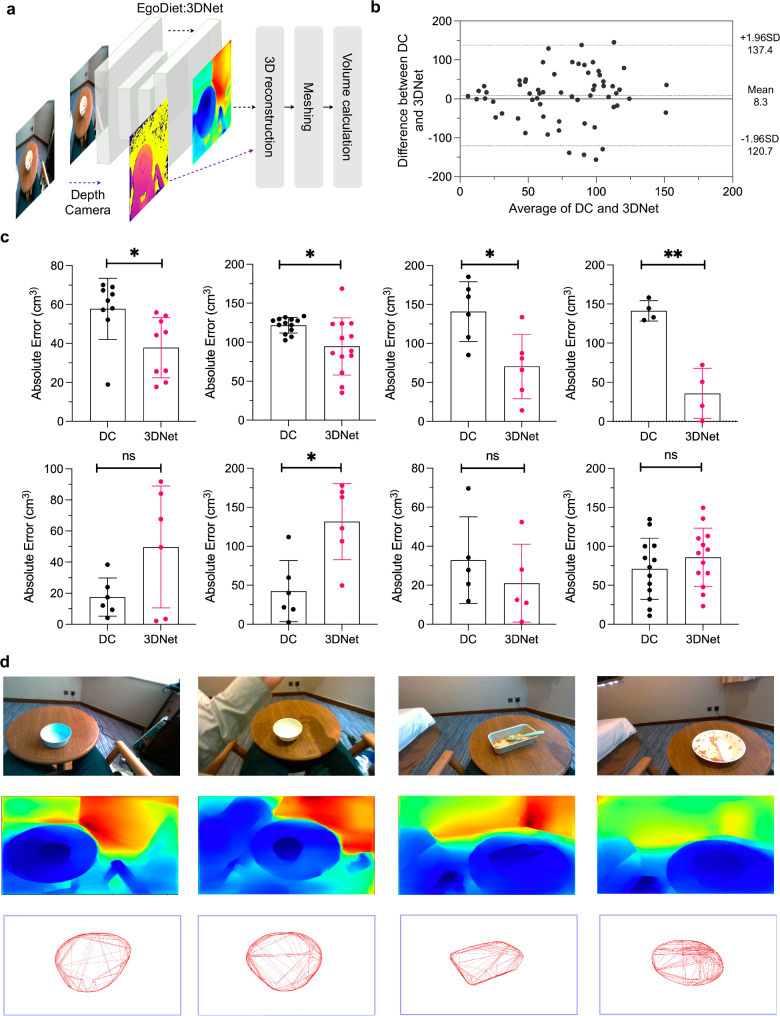
The performance of EgoDiet:3DNet. **a** This illustration diagram provides a representation of the EgoDiet:3DNet as applied to the captured RGB images within the EgoDIMCAP-Lab dataset. Depth images are first inferred and subsequently re-projected into world coordinates to generate 3D point cloud. This is achieved by utilising the depth values and the camera’s intrinsic parameters. Following this, a convex hull algorithm is applied to the 3D point cloud to construct a mesh, from which the volume is calculated.The same procedure is also applied to depth images captured directly by Intel RealSense D405 short-range Depth Camera (DC). **b** Bland-Altman analysis results. We compared the absolute errors in volumes calculated from depth images captured by the depth camera against the estimated volumes. The majority of data points fall within the Limits of Agreement (LOA), indicating similar error magnitude between the two methods. **c** A t-test was also conducted in this study, and the absolute error is shown for each of the food containers (i.e. 8 containers are evaluated) captured with various viewing angles. Note that the symbols * and ** denote a significance level of *p* < 0.05 and *p* < 0.01 respectively. The “ns" notation is used to indicate that there is not a significant difference between the means of the groups being compared. **d** Qualitative results of the EgoDiet:3DNet. The figure presents three sequential rows which show RGB images, inferred depth images, and 3D meshes of food containers respectively.

We first assessed the performance of EgoDiet:3DNet on our self-constructed dataset, EgoDIMCAP-Lab dataset^3^, with paired RGB and depth images of containers in various sizes (i.e. a key aspect of the EgoDIMCAP-Lab dataset is that the actual volume of the containers was measured using the water displacement method to serve as precise ground truth reference). The images were captured by an Intel RealSense D405 short-range Depth Camera (DC), a wearable depth camera that requires a connection to a PC for operation. We evaluated our method on 8 eating episodes. Depth images, both captured directly and inferred through the EgoDiet:3DNet framework, are re-projected onto the world coordinate system to facilitate volume estimation (Fig. 3a). The estimated volume was then compared to the ground truth volume. Within this process, EgoDiet:3DNet demonstrates a Mean Absolute Error (MAE) of 71.7 cm^3^(95%CI: 60.3 cm^3^−83.1 cm^3^), obtained by averaging the absolute error in samples from all eating episodes, which is comparable to the sensor-based approach that achieved MAE of 80.0 cm^3^(95%CI: 67.8 cm^3^−92.2 cm^3^) (i.e. In scenarios lacking fiducial markers with known size, leveraging depth camera technology for volume estimation emerges as a widely adopted solution to address scale ambiguity problem, offering a direct measure of object dimensions by capturing their 3D spatial data^13^). Bland-Altman analysis was conducted between the errors of DC and EgoDiet:3DNet methods relative to the ground truth (Fig. 3b). The majority of points fall within the limits of agreement, indicating that the differences between the errors of the two methods, when compared to the GT, are within acceptable bounds. A t-test was also conducted in our study (Fig. 3c), and the results have shown that the EgoDiet:3DNet achieves performance comparable to that of depth camera measurements in our task. Table 2 presents the quantitative results of the EgoDiet:3DNet’s performance on rough volume estimation across different eating episodes (i.e. actual volume of the food containers used in each episode is provided). These analyses show the potential of EgoDiet:3DNet in real-world settings, where it could serve as a simple and cost-effective alternative to methods that rely on depth sensors. For better understanding, qualitative results of EgoDiet:3DNet are shown (Fig. 3d). But so far, EgoDiet:3DNet can only provide a rough scale estimation (i.e. categorising sizes into tiny, small, medium, and large), and there are still several challenges that need to be explored, such as the differences in depth estimation between indoor and outdoor environments, lighting issues, occlusions leading to incomplete 3D reconstruction, errors generated during meshing when calculating volume and camera calibration issues. We also noticed that passively selecting the right images for volume calculation is challenging since there’s a discrepancy between images of food containers with and without food. Therefore, it’s advisable to present the empty container in front of the camera before serving food or after eating, during these specific time frames. Furthermore, using only close-up images for volume estimation can lead to significant errors in the depth estimation network. The capture process requires some environmental information, allowing the network to rely on its understanding of the surroundings to estimate distances. If standard-sized objects such as forks, spoons, or other items are also placed next to the container, it can further enhance the determination of the food container’s scale through calibration. To achieve higher accuracy or robustness, fine-tuning the depth estimation network with data from relevant test scenarios with known metric distances can lead to improved outcomes as well. With the emergence of large-scale foundation models in depth estimation, these processes have become relatively easier and their accuracy is gradually improving, opening up opportunities for more research in this direction.

**Table 2:** Evaluation of EgoDiet:3DNet’s performance on volume estimation of food containers with MAE and statistical metrics across varied eating episodes

Dataset	Eating episode #	Actual volume of food container (cm^3^)	EgoDiet:3DNet MAE ± SD (cm^3^)	EgoDiet:3DNet 95% CI (cm^3^)	Depth camera MAE ± SD (cm^3^)	Depth camera 95% CI (cm^3^)
EgoDIMCAP-Lab dataset	1	240	37.8 ± 15.4	[26.0–49.7]	57.8 ± 15.7	[45.7 - 69.9]
	2	375	94.6 ± 36.7	[72.4–116.8]	121.7 ± 10.0	[115.7 - 127.8]
	3	680	70.4 ± 41.2	[27.1–113.7]	140.9 ± 38.5	[100.5 - 181.2]
	4	500	35.8 ± 31.8	[−14.9–86.4]	141.3 ± 13.0	[120.6 - 162.1]
	5	375	49.7 ± 39.2	[8.6–90.9]	17.5 ± 12.3	[4.6 - 30.4]
	6	240	131.6 ± 48.9	[80.3–182.8]	42.5 ± 39.2	[1.3 - 83.6]
	7	240	21.0 ± 20.0	[−3.8–45.8]	32.8 ± 22.2	[5.2 - 60.4]
	8	500	85.9 ± 37.4	[63.3–108.5]	71.1 ± 39.2	[47.4 - 94.8]

Note that the volume estimation in this study utilises passive monitoring, which may potentially lead to a reasonable decrease in accuracy compared to active capture methods optimised for ideal capturing angles and distances.

#### Feature extraction module: EgoDiet:Feature

For the successful application of portion size estimation, it is necessary to ensure the features learned by the models are meaningful for the respective downstream tasks. The feature extraction module extracts important features from the previous modules. As mentioned previously, it is ineffective to estimate portion size based on a network trained end-to-end (i.e. using RGB images as input and corresponding portion size as output) due to insufficient training data. For this reason, our research group developed an easy-to-implement and robust strategy, which is to build the portion size estimation model on top of the semantic segmentation network (EgoDiet:SegNet) and depth estimation network (EgoDiet:3DNet) using task-relevant handcrafted features. These features were selected due to their direct relevance to the physical attributes of food portions that influence weight. With the use of the domain knowledge to constrain the learning algorithm, less representative training data is required, since the estimation does not rely on hidden implicit features anymore (i.e. entirely bypassing the requirement of large amount of training data for each food category in building a robust deep learning model). Another advantage is that the network generalises better and becomes more robust in handling unseen scenarios under free living which facilitates portion size estimation in different environmental conditions. The designed features include Food Type (FT), Food Region Ratio (FRR), Volume of Container (VC), Plate Aspect Ratio (PAR), Pixel Number of the Plate (NP) and Area Weight Ratio (AWR). FT is represented through one-hot encoding to categorise each food item into one of 15 types; FRR calculates the proportion of each food item within the container by comparing pixel counts; VC provides a rough estimate of container size, especially useful when no reference objects or depth sensors are available; NP offers an approximation of the distance between the camera and the food containers; PAR indicates the container’s height-width ratio to reflect the camera’s viewing angle; AWR refers to the weight per unit area, which is pre-determined through regression analysis (See 'Methods' for the detailed explanation for each designed feature).

#### Explainable AI identifies useful portion size-related features

**Fig. 4:**
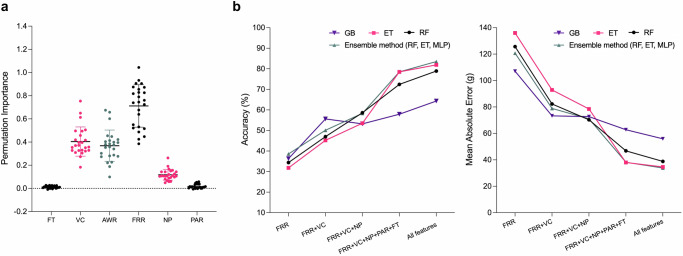
Analysing the importance of portion size-related features. **a** Permutation importance. We found that the food portion size estimation relies heavily on Food Region Ratio (FRR). The other features, including Food Type (FT), Volume of Container (VC), Pixel Number of the Plate (NP), Plate Aspect Ratio (PAR), Area Weight Ratio (AWR), have relatively less importance but still with positive effect on the estimation. **b** Evaluation was conducted using different portion size-related features and ML-based regressors, including Extra Trees (ET), Random Forest (RF), Gradient Boosting (GB) and the ensemble method. We reported the accuracy and MAE of the models. Among all these models, the ensemble method with full features obtained the best performance with higher accuracy and lower MAE compared to the rest of the models. Note that varying test data (i.e. such as capturing angles, container volumes, and the complexity of eating episodes) may lead to deviations in the results of different methods. Therefore, the ensemble method may not necessarily be the best-performing model in all scenarios. Nevertheless, our experiment clearly demonstrates the potential of the portion size-related features we extracted.

When developing a model to estimate food portion sizes, achieving accurate predictions of the food items’ weight is crucial. However, it’s equally important for the model to be interpretable. We need to comprehensively understand the factors leading the model to categorise the weight of food as high or low after analysing the images. Identifying the most significant features in determining weight estimations is a key part of this process. To facilitate this, we employ a technique known as Mean Decrease Accuracy (MDA), also referred to as permutation importance, on our self-collected dataset comprising 261 food instances. This dataset includes different types of food, various capturing angles and distances, and varying sizes of containers and food portions. This approach helps us find the significance of each feature we’ve proposed and discern their impact on the final estimation. Permutation importance is particularly useful for evaluating non-linear estimators. It disrupts the association between the target and individual features by randomly permuting them across various subjects, thus allowing us to assess the effect of each feature on the model’s accuracy. Fig. 4a shows that all features have positive permutation importance while a positive value corresponds to a deviation from the null hypothesis (i.e. null hypothesis refers to no statistically significant relationship between the feature and the portion size). In our analysis, it becomes evident that the model for estimating portion sizes is particularly reliant on the Feature Region Ratio (FRR), which has a mean importance score of 0.7 and accounts for 43.5% of the overall influence. Notably, the FRR is derived through our semantic segmentation module, showcasing the module’s integral role in calculating this feature. Beyond the FRR, the others offer relatively smaller contributions, their importance is still highlighted by the fact that they enhance the accuracy of portion size estimation when included, as evidenced by comparisons with models trained without these features (See Fig. 4b).

#### The robustness of EgoDiet:Feature

To verify the consistent performance of our proposed method in estimating portion sizes amidst natural body movements during meals, we assessed images captured from various viewing angles within identical scenarios. Our findings suggest that key features related to portion size, especially FRR, maintained consistency across different frames, which can be seen in the first and last row of Supplementary Fig. 1a. This consistency indicates that our method is robust in handling video footage with reasonable body movements. We also observed a significant difference in FRR between the second image in scenario 2 and the others, due to the food container being poorly captured, leading to overestimation of the portion size. This highlights the importance of using data pre-processing techniques in monitoring nutritional intake through passive methods. Another interesting finding is that when we compare the value of PAR in the first row of images with those in last row, we found that the former one is smaller. This is because the images in the first row were captured by a camera tilted downwards (i.e. similar to a top-down view). To further evaluate the performance of PAR, we performed an ablation study to classify whether the image was captured by eButton or AIM using PAR as the only feature (i.e. the images captured by AIM were tilted downwards, while the images captured by eButton were not). We trained a MLP classifier for this specific task and the final testing accuracy can achieve up to 98.5%. This shows a promising preliminary result on the challenging problem of estimating relative viewing angles of the cameras from images captured in the wild without relying on any deep learning-based approaches. These results unveil the potential of using the feature PAR (i.e. the aspect ratio of the food containers) to infer the viewing angles of wearable cameras, which in turn provide valuable insights into how to handle images captured with unseen positions.

#### Portion size estimation module: EgoDiet:PortionNet

Portion size estimation module refers to a network which is implemented by a stacking ensemble Machine Learning (ML) method by combining a Multi-Layer Perceptron (MLP) neural network with Random Forest (RF) and Extra Trees (ET) regressors. The module takes the designed features as input and outputs the estimated portion size, i.e. the output of EgoDiet:PortionNet is in grams since the ground truth weight (*g*) of a food item is easier to obtain than its actual volume (*cm*^3^). Several ML methods are also evaluated using the designed features to see whether these features also perform well in different ML models.

#### The validation of EgoDiet:PortionNet for portion size estimation

After the feature extraction phase, EgoDiet:PortionNet is employed to estimate the portion sizes of food items using the extracted features. The portion size estimations are then compared to WFRs, which serve as the ground truth. Following prior works, k-fold cross-validation (*k* = 15) was first used to examine EgoDiet:PortionNet. Figure 4b shows the accuracy of models that incorporate Machine Learning (ML)-based regressors, each trained with a combination of the proposed features pertinent to portion size estimation. The incremental addition of features results in improved accuracy for most ML-based regressors, underscoring the positive contribution of each feature to the accuracy of the final portion size estimation. Furthermore, we calculate the MAE of the portion size estimations to assess its performance. Distinct from previous studies that have utilised food volume error as the metric for evaluation^5,25^, this work adopts food weight error, expressed in grams, as a more intuitive metric for dietitians and a potentially more relevant measure for dietary assessments. Our models, when equipped with the full features, exhibit enhanced performance across most of ML-based regressors. Notably, the ensemble method emerges as the most proficient, achieving a MAE of 33.8 g. The performance of other ML models is shown in Table 3 for a detailed comparison. In Table 3, apart from feature inclusion studies, ablation studies demonstrate the impact of excluding specific features. The absence of FRR, FT/AWR, and VC corresponds to the evaluation of the segmentation module, food identification, and 3D reconstruction module, respectively. These studies aim to delineate the contribution of individual components to the overall performance of the system.

**Table 3:** Comparative performance of ML models with varying handcrafted feature sets in feature inclusion and ablation studies

	Handcrafted features						Accuracy (%)				Mean Absolute Error (MAE) (g)			
	FRR	VC	NP	PAR	FT	AWR	RF	ET	GB	Ensemble method	RF	ET	GB	Ensemble method
	*✓*						34.4	31.8	36.3	38.6	125.7	136.0	107.1	120.7
Feature inclusion studies	*✓*	*✓*					47.0	45.2	55.6	50.1	82.3	92.9	73.3	78.9
	*✓*	*✓*	*✓*				58.6	53.5	53.2	58.3	70.3	78.5	72.7	71.1
	*✓*	*✓*	*✓*	*✓*	*✓*		72.4	78.5	57.9	78.5	46.8	38.0	62.8	38.2
	*✓*	*✓*	*✓*	*✓*	*✓*	*✓*	78.9	81.9	64.3	83.5	38.8	34.6	55.9	33.8
Ablation studies		*✓*	*✓*	*✓*	*✓*	*✓*	60.6	58.6	54.0	62.5	65.1	66.1	73.5	64.2
	*✓*		*✓*	*✓*	*✓*	*✓*	74.3	76.6	64.7	78.1	46.0	40.0	58.4	39.8
	*✓*	*✓*	*✓*	*✓*			57.1	52.0	51.4	58.2	67.9	77.2	73.1	69.0

The methods for calculating Accuracy and MAE (Mean Absolute Error) can be referred to in the 'Methods' Section. *FRR* food region ratio, *VC* volume of container, *NP* pixel number of the plate, *PAR* plate aspect ratio, *FT* food type, *AWR* area weight ratio, *RF* random forest, *ET* extra trees, *GB* gradient boosting.

#### Comparison of portion size estimation by EgoDiet, weighed food record, and trained dietitians in semi-controlled study

**Fig. 5:**
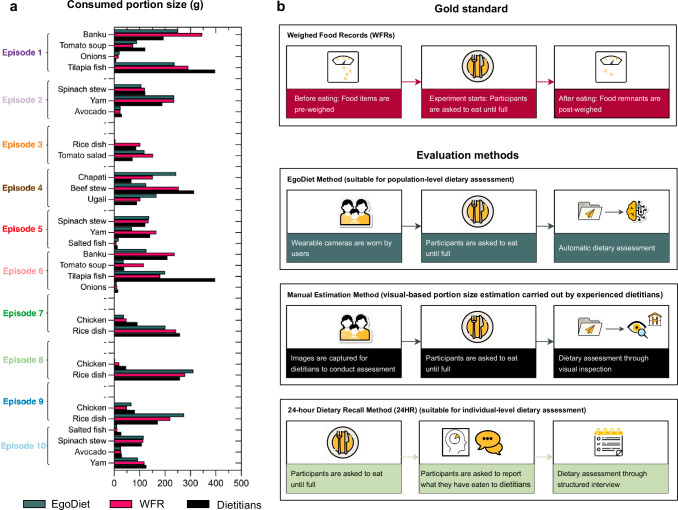
The validation of EgoDiet on portion size estimation in Study A. **a** The comparison between the EgoDiet and visual estimation by experienced dietitians on consumed food portion size across different episodes. Note that for bone-in foods such as chicken, we do not consider the weight of the bones in the consumed portion size in this study. Besides, given that Mask R-CNN may miss some small food items, for convenience in calculating the portion size estimation error, we do not consider the undetected food items. **b** An illustration diagram showing different evaluation methods used in our studies. In Study A, we compared our EgoDiet method with a manual visual-based portion size estimation method, while in Study B, we compared the EgoDiet method with the 24HR method, as described in the following section.

**Fig. 6:**
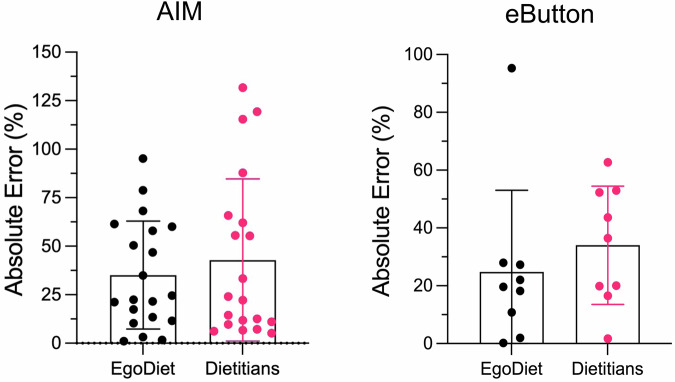
The comparison between EgoDiet and experienced dietitians on visual-based portion size estimation using AIM and eButton respectively. EgoDiet system using both AIM and eButton shows lower MAPE compared to dietitians' estimates.

Unlike food recognition, the existing research studies on portion size estimation (i.e. either food volume, food calorie or food weight estimation) have only examined their approaches on self-collected in-the-wild test datasets, in which there does not have an existing benchmark to conduct a fair comparison with previous methods. Thus, it is preferable to evaluate our proposed method in terms of practicality and implementation. To evaluate the practicality of the proposed system in assisting dietitians, we compared our model’s best performance (i.e. stacking method by combining MLP, RF and ET) (Fig. 2c) against experienced dietitians. Leave-one-out cross-validation was utilised in this study to ensure that the training data and test data did not come from the same video. We first estimated the net difference of the initial and final food weight (i.e. the consumed food portion size) using our pipeline EgoDiet. Raw video footages of 10 selected eating episodes in Study A were provided to the trained dietitians using the custom software (i.e. AIM software). The consumed portion size for each food item was estimated visually by the dietitians from the videos (Fig. 5b). Each video contains approximately 200–400 images per eating scenario. Note that the participated dietitians are familiar with the local food items and have extensive knowledge of visual-based portion size estimation. Finally, the estimation from EgoDiet is compared to the visual estimation by the dietitians (Figs. 5a and 6). To better visualise the performance, we report the MAPE of the consumed portion size for the EgoDiet and dietitians’ visual estimation. Our findings indicate an advantage for the EgoDiet system, which demonstrates a MAPE of 31.9%(95%CI: 21.8–42.1%), in contrast to the visual estimates by dietitians, which showed a MAPE of 40.1%(95%CI: 26.9–53.3%). In Fig. 6, we also present the performance of EgoDiet using AIM and eButton compared to dietitians separately. The MAPE observed with the AIM reaches 35.1% while deploying the eButton results in a MAPE of 24.8%. Note that the MAPE is calculated using WFRs as the ground truth in this study. Once the consumed portion size is obtained, we can use a regional nutritional database or the USDA nutritional database as a reference to derive the nutritional content (i.e. This step, demonstrated in Supplementary Table 2, 3 and 4, serves as an example of the potential applications).

#### Performance of EgoDiet in low-and-middle-income countries (LMICs)

**Fig. 7:**
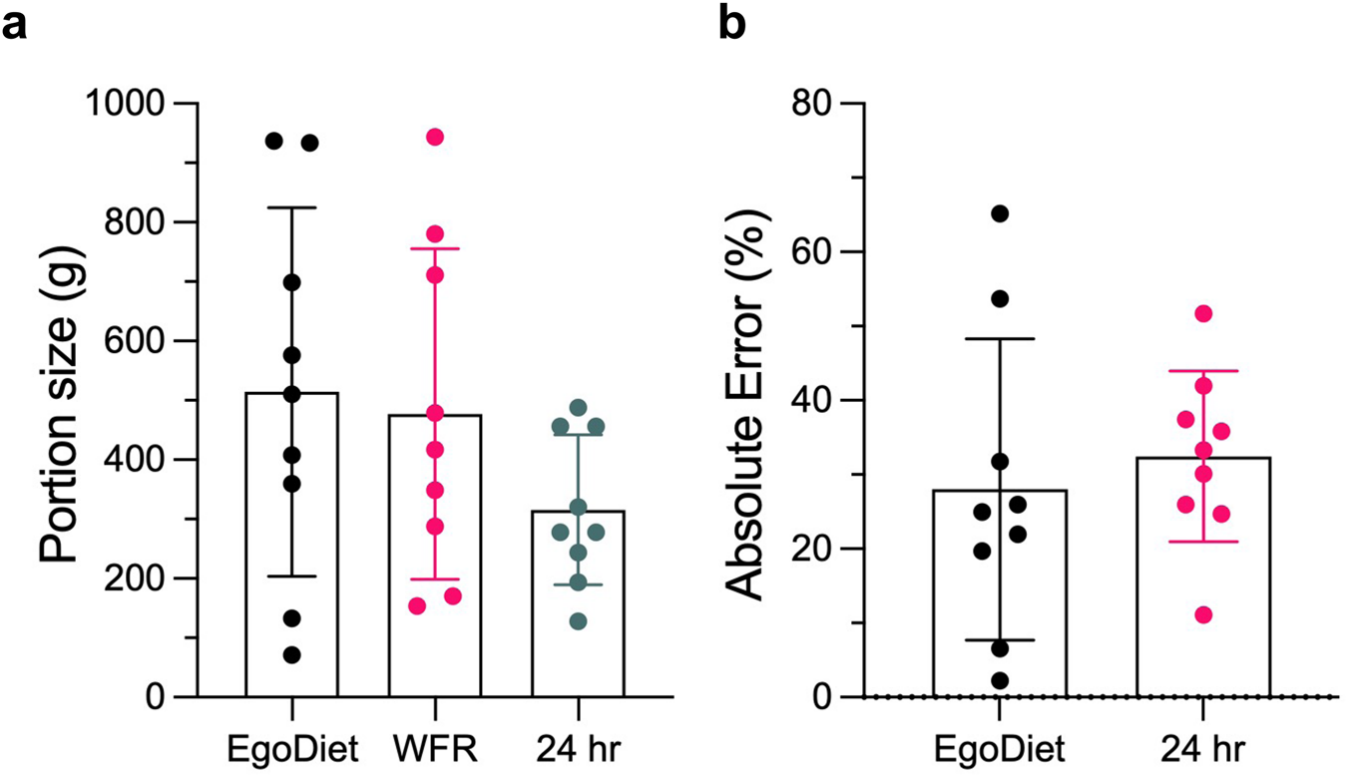
The comparison between EgoDiet and a commonly-used dietary assessment method, 24HR, on portion size estimation using data in the field site—Ghana. **a** The portion size reported/estimated by 24HR and EgoDiet. In the scatter dot plot presented, the mean and Standard Deviation (SD) are shown. It can be observed that the EgoDiet method and Weighed Food Records (WFRs) have similar means in portion size, indicating the potential usefulness of EgoDiet in assisting with dietary assessment. **b** Absolute error (%) on portion size estimation between EgoDiet and 24HR. Note that WFRs are used as the ground truth in this study.

To further examine the practicality of EgoDiet under free-living conditions, the wearable cameras, including the AIM and eButton (Supplementary Fig. 3), were dispatched to the volunteer households in the rural region of Ghana. Video footages were captured by the passive wearable cameras under free-living environment with minimal human intervention. In this study, we further trained our networks with augmented data and compared the performance of portion size estimation using EgoDiet with 24HR, a commonly used self-reporting dietary assessment method in nutritional epidemiology studies (i.e. the food types and the portion size are recalled from the participants and report to the experienced dietitians). WFRs were also used as the ground truth in this study (Fig. 5b). In Study B, each individual household prepared their own food at home; thus there were different varieties of food items among different households. To ensure a fair comparison, we only evaluated eating episodes that involved similar food categories as those in Study A (i.e. the seen/related food types). However, we also excluded some instances of consuming similar food types due to limitations caused by the orientation of the wearable camera, which may have impeded the capture of high-quality images necessary for accurate dietary assessment. Finally, our system was evaluated on 9 eating episodes. Figure 7a shows the consumed portion size reported and estimated by 24HR and our proposed EgoDiet respectively. We found that the EgoDiet achieves MAPE of 28.0%(95%CI: 14.8–41.3%), while 24HR achieves MAPE of 32.5%(95%CI: 24.9–40.0%) for portion size estimation (See Fig. 7b) (i.e. the MAPE is calculated using WFRs as the ground truth in this study). The results of Study B were found to be comparable to those of Study A. However, it should be noted that Study B was conducted in a more complex environment than Study A. The promising performance can likely be attributed to the fact that the food containers used in Study B typically contained only one type of food, which simplified the task. The outcome suggests that our proposed system is capable of achieving human-level performance in estimating food portion sizes, demonstrating its potential as an alternative to 24HR. This advancement could reduce the workload of dietitians by streamlining the dietary assessment process through the use of low-cost wearable cameras. Some samples of the experimental results obtained through the EgoDiet:3DNet, with the details of 3D reconstruction of food containers presented for different eating scenarios are shown in the supplementary material (See Supplementary Fig. 4).

## Discussion

In this study, we have proposed EgoDiet, a passive dietary monitoring system that uses AI and wearable technology to facilitate dietary assessment. We found that the system shows potential in reducing error rates in dietary assessment compared to traditional methods, suggesting it could be a promising alternative for monitoring dietary intake. Given the link between diet, non-communicable diseases, and associated health risks, there is a necessity for reliable methods to monitor dietary intake. The inaccuracies in reporting dietary intake based on the current self-reporting methods significantly undermine the integrity of various research outcomes, ranging from clinical trials and epidemiological studies in evaluating the impact of nutritional policies. Moreover, the burdens associated with the requirement to self-report diet on multiple occasions can affect eating behaviour, study recruitment, and importantly, subsequent retention, thus biasing participant demographics. However, there are a few methods available nowadays that can accurately monitor dietary intake for individuals in their own environments.

With EgoDiet, we addressed several challenges in the field of dietary assessment such as self-reporting bias and participant burden. In terms of practicality, EgoDiet enables dietary assessment using low-cost passive wearable cameras, which minimise the burden and technical requirements for dietary information collection and analysis. Our empirical analysis comprises three phases, including an initial controlled environment testing phase, followed by Study A (conducted in London) and Study B (conducted in Ghana). The controlled environment testing phase allowed us to assess the system’s performance under optimal conditions, establishing the accuracy and consistency of EgoDiet’s core functionalities. Building upon these foundational results, in Study A, we found that the error rate when using the AI-powered EgoDiet approach was 31.9% for portion size estimation, whereas with traditional dietary assessment by dietitians using visual estimation, the error rate was higher, at 40.1%. In Study B, we compared EgoDiet with another traditional method, the 24HR. The results show that the error rate for EgoDiet was 28.0%, while the error rate for the 24HR method was slightly higher, at 32.5%. These comparative analyses show the potential of passive wearable technology in capturing dietary intake information. Notably, traditional dietary assessment techniques, predominantly reliant on self-reporting mechanisms such as 24HR and food frequency questionnaires, have been noted for their difficulties in achieving high levels of precision. Previous studies^26,27^ have highlighted the significant challenge of under-reporting, with rates reaching up to 59%, raising concerns about the reliability of these conventional methods. Besides, with our technology, dietitians are not required to conduct structured interviews to gather information on food types and portion sizes, which can reduce their workload, especially during large-scale dietary assessments. Furthermore, the cost of wearable cameras is comparable to conducting a single 24HR. As cameras are mass-produced, the cost can become even more favourable. Additionally, the reusability of cameras offers an additional cost advantage for our technology. The use of low-cost wearable cameras, which can be distributed and used by multiple participants simultaneously, also accelerates the speed of data collection.

From a technical perspective, to minimise the impact on participants’ normal behaviour when using our technology, we do not consider using any fiducial markers, but it indeed makes the task particularly challenging due to the scale ambiguity. To address this issue, we proposed EgoDiet:3DNet, a model that could roughly estimate the scale of food containers. This demonstrates the potential of employing standard RGB cameras as a substitute for costly depth cameras, offering an alternative solution to the portion size estimation. Despite the advancements in learning-based depth estimation methods, there are still constraints due to the requirement for training and testing within similar scenarios. As a result, these models are not sufficiently generalised, which can lead to less accurate estimations in significantly different scenes. Nonetheless, the continuous evolution of AI techniques is progressively addressing this challenge. Furthermore, EgoDiet:Feature and EgoDiet:PortionNet are engineered to operate together, enabling accurate portion size estimation with few annotations. With the use of the domain knowledge to constrain the learning algorithm, our network can directly target the portion size-related features without requiring to extract implicit features from large volume of training data (i.e. paired RGB images and their corresponding portion size cannot be easily obtained). This multi-module approach to calculating portion size, while potentially leading to cumulative errors, also has its advantages. It allows for the timely replacement of previous modules with state-of-the-art (SOTA) ones. For instance, with the rising popularity of large-scale foundation models, the accuracy of recognition (e.g. LLaVA^28^, GPT-4V), segmentation (e.g. Segment Anything^29^), and depth estimation (e.g. Depth Anything^30^) is continually improving, enabling us to optimise the performance of our network. Last but not least, it’s important to note that the majority of current algorithms are tailored for active food capture, which poses a challenge for integration with passively capturing wearable cameras. Techniques such as stereo-based methods, model-based approaches, and perspective transformation all come with their own limitations. These include restrictive capturing angles, dependency on distinct features on food items, and a need for user intervention. Such constraints significantly impede the scalability of dietary assessments.

However, there are also limitations and areas for improvement in our system, such as the privacy issues brought about by passive monitoring, which require corresponding measures. Currently, our approach involves extracting and filtering images that contain food or food containers^3^. During this step, any images that do not meet these criteria, including those that may capture private activities (such as using the bathroom), are automatically identified and excluded by the AI. The second step involves blurring faces in the remaining images to ensure anonymity. This two-step approach ensures that sensitive information is filtered out early in the process, providing an additional layer of privacy protection. Although AI image extraction and blurring have already matured and effectively address privacy issues, there remains potential for optimization. It continues to be a key focus for the development of passive monitoring devices. Moreover, our passive monitoring system has yet to achieve the accuracy of active capturing methods in estimating portion sizes^31,32^. This is primarily due to challenges such as the inability to consistently capture food containers at the image centre due to natural body movements and hand occlusion, as well as difficulties in targeting the specific start and end time frames required for calculating consumed portion sizes. These aspects underscore opportunities for further improvement within our system.

For future work, our focus will be on enhancing the food recognition capabilities of the model to make it more general and capable of identifying a wider range of food categories. Currently, our model focuses on broader food categories rather than fine-grained types. In the future, we plan to extend the system to recognize more detailed and specific food items, allowing for a finer analysis of nutritional content. Moreover, the robustness and precision in the estimation of portion sizes warrant significant enhancement. The integration of large-scale foundational models presents a promising avenue for using contextual cues from the surrounding environment to deduce food portions more effectively. Another challenge in the field of dietary assessment is the difficulty associated with shared plates^33^, as well as distinguishing between the acts of consuming and preparing a meal through passive monitoring. To address these problems, we are going to incorporate learning-based action recognition methods to identify various daily activities, thereby augmenting the intelligence and accuracy of our system. Apart from software improvements, we are also focused on hardware enhancements. In response to factors like image quality and capture angle of the devices, we are actively working on hardware upgrades, such as enhancing resolution and expanding the field of view, while ensuring that the capture angle remains consistent when participants wear the device. Beyond the technical scope of future work, the inherent characteristics of passive monitoring enhance the range of data available for analysis in this domain. Passive monitoring enables the capturing of eating sequences, the identification of food items left unconsumed by the participants, and the record of meal timing and duration, from which the habits and eating behaviours of the participants can be deduced. Such data could offer significant potential for future research in nutritional epidemiology. Overall, we believe that EgoDiet represents a valuable contribution to the field and has the potential to assist dietitians in their work to promote healthier diets.

## Methods

### Ethics approval

The Imperial College Research Ethics Committee has provided ethics approval for the studies: approval reference number is 18IC4780 for the study at the CRF; The studies for Ghana have been approved by the ethics committees (Institutional Review Boards) at both the University of Georgia, USA, and the Noguchi Memorial Institute for Medical Research, Ghana. The ethics approval references are STUDY00006121 and IRB00001276.

### System modelling

The wearable cameras will be distributed to the participants, set up prior to the study and recollected after the study by our staff in the field sites. However, the urban-rural divide in LMIC could complicate the implementation of the system. In consideration of the low internet availability and inadequate electricity supply in rural regions, we temporarily store the captured images to the on-board SD card, and collected by our staff and uploaded to the cloud server after returning to urban regions. After uploading the images, the images will be forwarded to the trained networks for dietary assessments (i.e. testing process). Note that all participants have provided written consent to participate in the study.

### Raw video footage processing

Despite the convenience of the passive method, the implementing strategies are relatively complicated, since the data storage for continuous long-term surveillance is a challenging technical issue. It is because the passive method will capture image frames continuously, which requires a huge memory space of the devices and extensive amount of time for dietitians to manually scan through the images to estimate individual dietary intake. Several previous studies proposed selecting representative frames by circular plate detection using Canny detector^18,34^. Recently, the work^35^ proposed using Clarifai CNN, which outputs tags for each input image and determine the importance by counting the food-related tags. Similar to the work^35^, we also proposed a deep learning method to determine whether food items are presented in the images or not in our recent work^3^. Our proposed method is designed to add captions to each captured image, thereby facilitating the identification process. If the images display captions with food tags, they are classified as food-related frames. We also proposed a classification method to further remove images where the full food container is not visible (i.e. images with incomplete food containers). These removals are critical as our proposed portion size estimation network relies on the proportion between the food container and the food items on it. Conducting the estimation on images where only parts of the food container are visible increases the proportion of food items which in turn causes overestimation of the food weight. In developing the method, we first generated the segmentation masks of the food containers for each image using Mask R-CNN. Then an alterable edge region was defined, of which the parameter (e.g. the width of the edge region) could be changed according to the resolution of the images captured by wearable cameras (e.g. 640 × 480, 1280 × 720). When a high proportion of the area by the segmentation mask of the food container and edge region is overlapped, the corresponding image will be considered as containing an incomplete food container (Fig. 2d). Besides, privacy and security are of utmost importance in passive monitoring system, before uploading any images, we apply AI-driven blurring to anonymise faces and each image is encrypted to prevent access by non-research personnel.

### 3D reconstruction of food containers

To estimate the volume of a food container, we utilise a depth estimation network with encoder-decoder architecture^3^ to determine the depth of an RGB image featuring an empty container (i.e. the use of relevant regional scene images in transfer learning leads to a more substantial improvement in performance). We then project the estimated depth image into 3D space, creating a 3D point cloud. From this, we extract the 3D point cloud of the container and apply the 3D convex hull algorithm to calculate the container’s volume. The convex hull is the smallest possible convex set that contains all the points of a 3D model. For more information on volume calculations, please refer to our previous works^5,36^. The volume measured for the empty food container has the potential to provide scaling information, thereby aiding in the calculation of the actual portion size of the food consumed. Note that the selection of images for estimating the scale of food containers is not yet fully automated and involves certain human intervention. Selecting images without hand obstructions and containing empty containers improves the performance of 3D reconstruction and enhances the accuracy of scale determination. Additionally, including general objects of known or standard sizes, such as forks and spoons, in the selected images as references could aid in the calibration process.

### Semantic segmentation training, hyper-parameter settings and dataset preparation

The semantic segmentation module used in our study is based on the Mask R-CNN^2^, pre-trained on the COCO dataset^37^. The network architecture of Mask R-CNN is designed to perform both object detection and instance segmentation, leveraging various backbone architectures for feature extraction, bounding box recognition and mask estimation. The Mask R-CNN architecture can be divided into several parts including the backbone architecture, Region Proposal Network (RPN), RoIAlign, object detection branch and mask generation branch. The backbone architecture employs ResNet-101^38^ in conjunction with the Feature Pyramid Network (FPN)^39^ for feature extraction. FPN constructs a multi-scale feature pyramid, leveraging a top-down architecture with lateral connections. This design enables the extraction of Region of Interest (RoI) features at various levels of the pyramid, adapting to their scale. The following Region Proposal Network (RPN) is then used to generate region proposals that are likely to contain objects. Object detection branch processes aligned RoI features to classify objects and refine their bounding boxes. It identifies the object’s class and adjusts the RoI to closely match the object’s location and size. Working alongside the object detection branch, mask generation branch predicts a pixel-level mask for each detected object in a RoI. For further details on the implementation of the Mask R-CNN architecture (pre-trained on the COCO dataset) and hyper-parameter settings used in this study, we refer readers to the work by Abdulla et al. on GitHub^40^. Due to the differences between the food categories in the COCO dataset and the African food items, the pre-trained model can only segment a limited number of food categories. This is not sufficient to develop a dietary assessment system for African populations. Therefore, 15 common African food items were selected for annotation: onions & tomato salad, tilapia fish, ugali, yam, chicken drumstick, spinach stew, avocado, banku, tomato soup, roasted beef, chapati, onions, rice dish, salted fish, and beef stew, using the VGG Image Annotator (VIA), following the procedures described in our previous publication^33^. Food containers such as plates and bowls were individually categorised and trained to aid both data preparation and portion size estimation. These annotated images were then combined with the Diabetes60 dataset^23^. Data augmentation was used to increase the diversity of the dataset for training the models, in which flipping, rotating, shearing and cropping were applied to the input images. Mask R-CNN can accommodate input images of varying dimensions, and resizing is not required before feeding them into the network. All the networks were trained using the Adam optimiser on an NVIDIA GeForce RTX 2080 graphics card, utilising Python with Keras and TensorFlow frameworks. The best-trained model was then applied to the images after feature extraction. The loss function of the semantic segmentation module is shown in Equation (1):

1 $${L}_{total}={L}_{cls}+{L}_{box}+{L}_{mask}$$

where *L*_*cls*_, *L*_*box*_ and *L*_*mask*_ refer to the loss of classification, localisation and segmentation mask respectively. *L*_*cls*_ and *L*_*box*_ are the same as in traditional object detection models. *L*_*mask*_ can be represented as the average binary cross-entropy loss and its formula for food category *k* is described in Equation (2).

2 $${L}_{mask}=-\frac{1}{{d}^{2}}\sum _{1\le i,j\le d}[{y}_{ij}log{\hat{y}}_{ij}^{k}+(1-{y}_{ij})log(1-{\hat{y}}_{ij}^{k})]$$

where *y*_*ij*_ is the ground-truth label of the pixel(i,j) in a region of size *d* × *d* and ${\hat{y}}_{ij}^{k}$ is the predicted label of the corresponding pixel learned for food category *k*. When the loss function *L*_*total*_ is minimised, the segmentation masks of food items and containers are generated. By doing so, the segmentation masks help extract features for each particular food item and facilitate consumed food weight estimation for the following procedure. Since the focus of this paper is not on food segmentation, Mask R-CNN was not subjected to separate evaluation. For the effectiveness of Mask R-CNN in food segmentation, we have explored this in our previous publications^33,41^.

### Feature extraction for portion size estimation

The detailed information of the portion size-related features is summarised as follows:

- *Food Type (FT)*: One-hot encoding is applied in which each categorical feature is encoded as a one-hot numeric array. It is represented by a vector with all 0 except one, which has 1 as value in its corresponding food category (15 × 1 vector in this case due to 15 food types).
- *Food Region Ratio (FRR):* This feature refers to the ratio of the pixel number of each individual food item to plate (*NF*_*i*_/*NP*), which gives an indication of the proportion of the region for each food item in the container.
- *Volume of Container (VC):* This feature provides a rough estimation of the container size. By applying EgoDiet:3DNet to the eating episodes, we can obtain the volume of the container. A more accurate estimation results from averaging the volumes derived from multiple images. Considering the reasonable inaccuracies in estimating container volume under free-living conditions, we round this output to the nearest 200 cm^3^. Although this is not highly precise at the moment, it can provide a general indication of distinguish whether the container is tiny, small, medium, or large (i.e. categorical volume calculation). Additionally, if the food containers in both the training and test datasets have the same or similar dimensions, this feature can be disregarded to simplify the training process.
- *Pixel Number of the Plate (NP):* This feature gives a rough estimation of the distance between the cameras and the food containers when the food containers used in both the training and test dataset are the same.
- *Plate Aspect Ratio (PAR):* This represents the height-width ratio of the container, which gives an indication of the camera viewing angle. PAR can be derived using the coordinates of the plate’s segmentation masks based on Singular Value Decomposition (SVD), as shown in the following equation and in Supplementary Fig. 2. The idea of PAR is quite intuitive. Consider two scenarios: one where an image is captured directly from above and another from an angle. Under EgoDiet’s SegNet, these perspectives would yield different segmentations, altering the proportion of the food mask within the container for the identical eating episode. This leads to variations in the FRR, potentially introducing errors in estimating portion size. It’s also important to note that variations in dish size do not affect the PAR, as this ratio is calculated based on the length-to-width proportions of the plate. Assuming the dishes are captured from a direct overhead perspective, the dimensions of the dish’s length and width are represented consistently in the images, thereby maintaining a consistent PAR across different dish sizes.where the columns of *U* and the rows of *V*^*T*^ are the left singular vectors and right singular vectors respectively. *Σ* is a diagonal matrix which presents the singular values. Then the *PAR* can be calculated by $\frac{{\sigma }_{1}}{{\sigma }_{2}}$.
- *Area Weight Ratio (AWR)* Different food items have their corresponding geometry as well as density (g/cm^3^), and these features will affect the food weight estimation. However, from a single image without relying on any depth information, it is often difficult to infer the full geometry of the food items. Thus, AWR feature is designed for rough portion size estimation using a single image. Each food item has its corresponding AWR value, e.g. Beef Stew: 2.3; Yam: 3.5; Avocado: 2.0, which represents the amount of weight per cm^2^ (g/cm^2^). This value can be determined through regression for each particular food item before the experiments (i.e. draw a plot of mean surface area (cm^2^) versus weight (g) of each food item).

### Data augmentation in portion size estimation model

In this study, we utilise a domain-specific data augmentation technique known as volume-weight scaling method, which leverages the physical relationship between the inner volume of a food container and the weight of the food it contains. After applying EgoDiet:3DNet and EgoDiet:Feature to a specific image frame, a set of portion-size related features can be obtained, which form a training pair when coupled with food weight. By tuning the VC, we can generate additional training pairs as the food weight varies linearly with the inner volume of the containers. This technique is particularly useful since it has the potential to estimate portion sizes for food containers with previously unseen sizes, expanding the applicability of our method. Exploring enhanced data augmentation methods for the task of portion size estimation is an interesting topic for future research.

### Implementation of ML models

In our study, we implemented a variety of machine learning models using Python, relying on the scikit-learn library for the implementations. We created several models, each with explicitly selected hyperparameters to optimise its performance. We employed random forest, extra trees, and gradient boosting regressors, configured with 10, 10, and 20 estimators, respectively. Lastly, our ensemble approach involved a stacking regressor, where we stacked a random forest regressor and an extra trees regressor, each with 10 estimators, as base estimators. The meta-regressor in this ensemble was a Multi-Layer Perceptron (MLP) regressor, detailed with hidden layer sizes of (250, 250, 250, 250, 125, 50), ’relu’ activation, ’adam’ solver, an ’adaptive’ learning rate, and a maximum of 10k epochs, ensuring thorough convergence of the model.

### Evaluation metrics

We assessed the performance of the proposed models using several evaluation metrics, including permutation importance, accuracy of the portion size estimation, MAPE.

3 $${i}_{k}=s-\frac{1}{M}\mathop{\sum }\limits_{m=1}^{M}{s}_{m,k}$$

where *i*_*k*_ refers to the permutation importance for feature *k* and *M* refers to the number of repetition (*M* = 20). *s* represents the reference score of the model evaluated on raw dataset, while *s*_*m*,*k*_ refers to the reference score of the model evaluated on the shuffled dataset (for feature *k*).

4 $${\text{Accuracy}}\,=\frac{1}{M}\mathop{\sum }\limits_{i=1}^{M}f(||{y}_{i}-\hat{{y}_{i}}|| \,<\, \epsilon )$$

where *f*(true) = 1 and *f*(false) = 0 is a Boolean function, and *ϵ* is the tolerance for determining a correct prediction (*ϵ* = 60). *M* refers to the total number of test data, $\hat{{y}_{i}}$ and *y*_*i*_ represents the ground truth and the estimated weight for the *i*th sample respectively (i.e. visual estimation of portion size is generally limited in precision, and small errors in weight(g) may be difficult to detect).

5 $${\text{MAPE}}\,=\frac{1}{M}\mathop{\sum }\limits_{i=1}^{M}\left|\frac{{y}_{i}-\hat{{y}_{i}}}{{y}_{i}}\right|\times 100$$

where *M* is the total number of samples, *y*_*i*_ is the ground truth value for the *i*th sample and $\hat{{y}_{i}}$ is the predicted value for the *i*th sample. The absolute difference between the ground truth and predicted values is divided by the ground truth value and multiplied by 100% to obtain the percentage error for each sample. The average of these percentage errors across all samples gives the mean absolute percentage error. In our study, MAPE is indeed a metric used to calculate the error in consumed portion sizes. *y*_*i*_ refers the value of WFRs for the *i*th food item and $\hat{{y}_{i}}$ is the estimated consumed portion size by EgoDiet or dietitians.

## Supplementary information

Supplementary Material

## Data Availability

The dataset cannot be made publicly available due to privacy protection, general data protection regulations, and institutional guidelines of the Noguchi Memorial Institute for Medical Research Institutional Review Board (NMIMR-IRB).

## Code availability

The AI model was implemented using Python 3, Keras, and TensorFlow. The code for segmentation is available at https://github.com/matterport/Mask_RCNN. The machine learning algorithms can be implemented using the sklearn library.
