# Supplementary material for: AI-enabled wearable cameras for assisting dietary assessment in African populations
Source: NPJ Digit Med. 2024 Dec 5;7:356. doi: 10.1038/s41746-024-01346-8 (PMC11621677; doi:10.1038/s41746-024-01346-8)
Supplement: Supplementary file 1 — Supplementary Material [file 41746_2024_1346_MOESM1_ESM.pdf]

Supplementary Material

Supplementary Table 1. Descriptions of recent research works utilising passive methods in dietary assessment

Recent works	Methods	Descriptions	Discussions
Qiu et al.[1] (2020)	Using a wearable camera (GoPro Hero 7) mounted on a subject's shoulder to capture the entire eating episode, followed by deep neural networks to process captured videos	Capable of estimating the number of bites taken and recognizing the food items consumed directly from a passively captured video	The current study examines the proposed approach in a laboratory setting only, and does not estimate the size of each bite, which is essential for quantifying dietary intake if a bite counting approach is to be adopted
Bahador et al.[2] (2021)	An efficient fusion method based on 2D covariance map of various wearable sensory signals such as IMU, followed by a deep neural network for activity recognition	Capable of distinguishing eating from other activities such as sleeping	The current study does not go further into detecting fine-grained eating activities such as chewing, swallowing, and cannot recognise food items and estimate food consumption
Doulah et al.[3] (2022)	Using a multi-modal wearable sensor called Automatic Ingestion Monitor 2 (AIM-2) to estimate Energy Intake (EI)	AIM-2 is able to estimate EI of meals from chewing sensor signals and food images	Having difficulty in estimating portion size, which results in errors in overall EI estimation
Elbassuoni et al.[4] (2022)	Using a deep learning model to evaluate the healthiness of food items	Capable of categorising food items into NOVA groups: unprocessed food, processed culinary ingredients, processed food, and ultra-processed food	Utilising a qualitative approach over a quantitative one, the method does not estimate the calorie/ portion size directly
Elbassuoni et al.[5] (2023)	Using a wearable camera with machine learning based data collection system to objectively capture school-children's exposure to food (real-world case study)	Capable of classifying food-related images into images that contain actual food items, images that contain food advertisements, and images that contain food outlets; Capable of classifying whether the food items are being consumed by the users or whether they are consumed by others	Requiring a very large training dataset, the labelling of which demands considerable effort; Depending on search engines for data labelling could lead to inaccuracies in the assigned labels

A more comprehensive descriptions and discussions of research works using passive methods in portion size estimation can be found in recent works[6, 7]

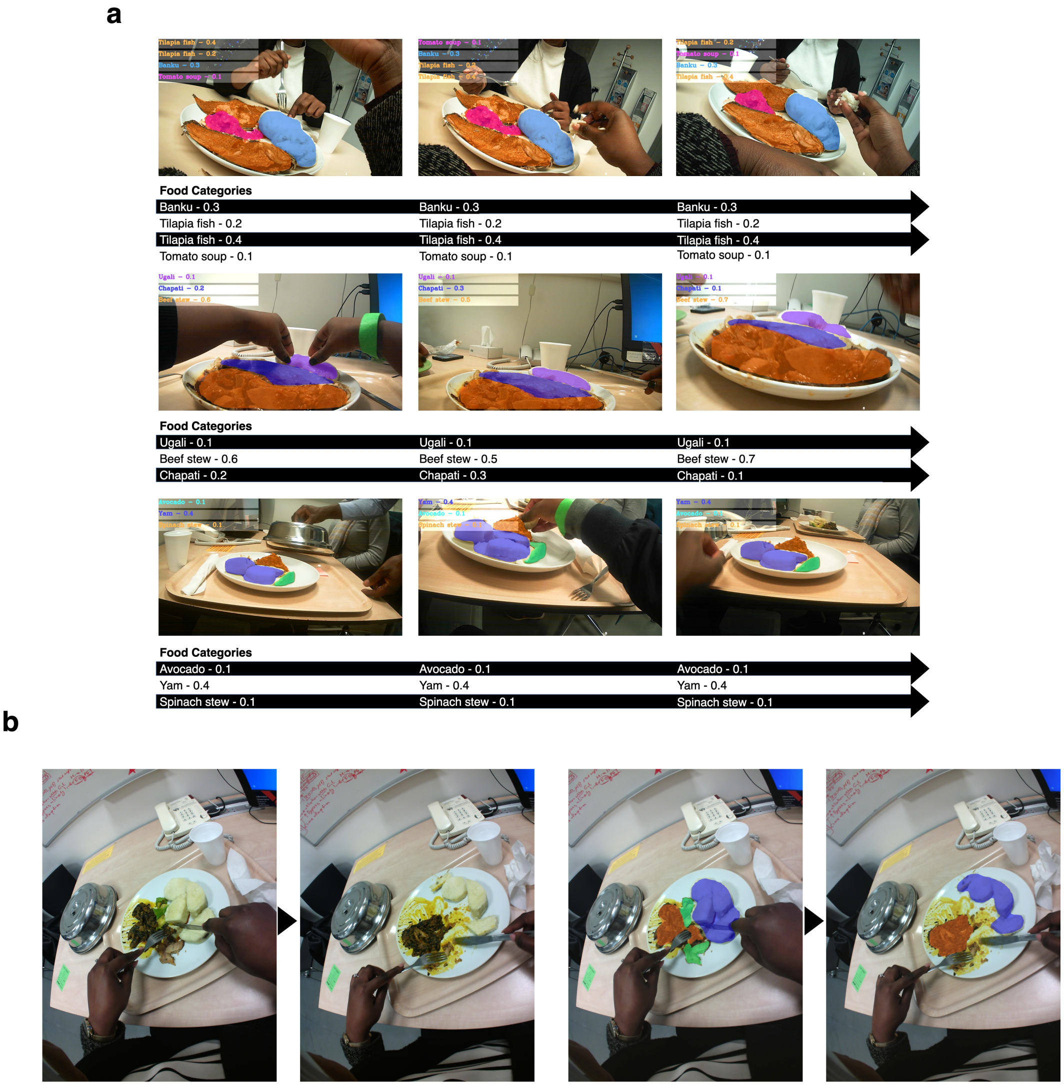

Supplementary Figure 1. EgoDiet:SegNet on semantic segmentation. (a) Illustrative examples obtained through the semantic segmentation module, with the details of handcrafted features presented for each frame; FRR (top left), PAR (top right). FRR value indicates the proportion of the region for each food item in the food container. We noticed that the FRR value for certain food item is overestimated when the captured images have incomplete container (in the middle of the second row), while the extracted FRR values are consistent for perfect containers even with postural instability. **(b)** Some samples of the eating scenarios captured by the AIM device with their corresponding segmented masks.

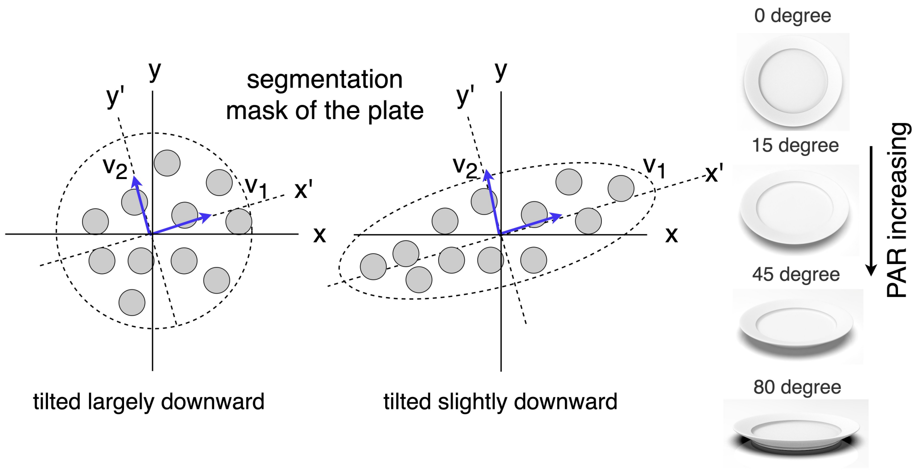

Supplementary Figure 2. The rationale of generating the feature - PAR. Singular Value Decomposition (SVD) on the segmentation mask of the plate (left) the camera is tilted largely downward (e.g., top-down view) (right) the camera is tilted slightly downward (e.g., more horizontal). Note that the PAR value will increase with the viewing angle as shown in the figure.

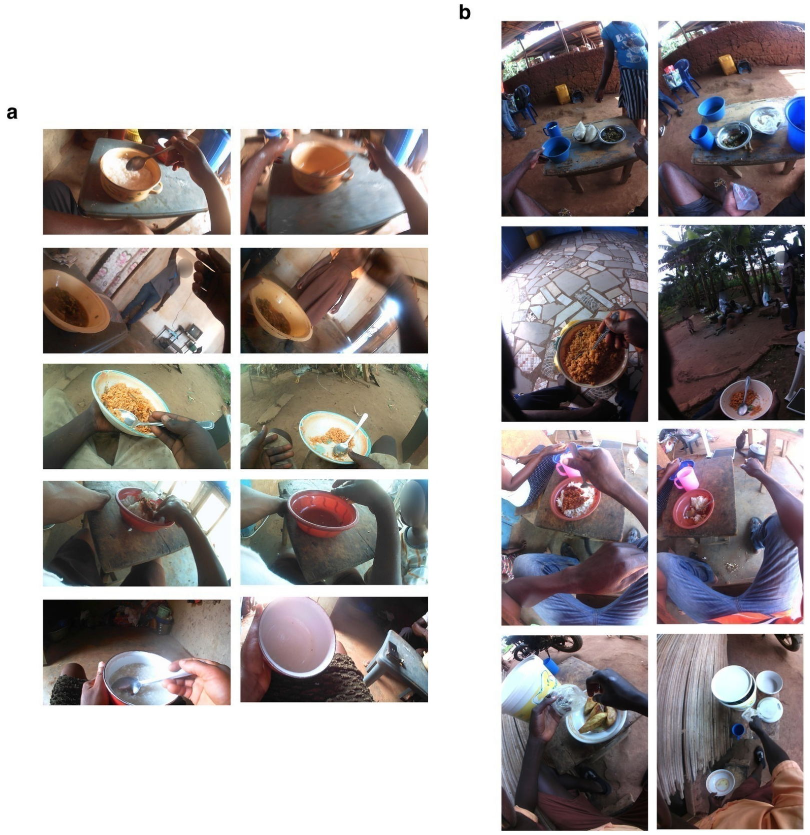

Supplementary Figure 3. Images of various eating scenarios captured under free living in the rural region of Ghana
Samples showing the starting and finishing time of different eating scenarios obtained through passive wearable cameras **(a)** the eButton and **(b)** the AIM respectively. Certain images are illustrative examples and are not included in the analysis.

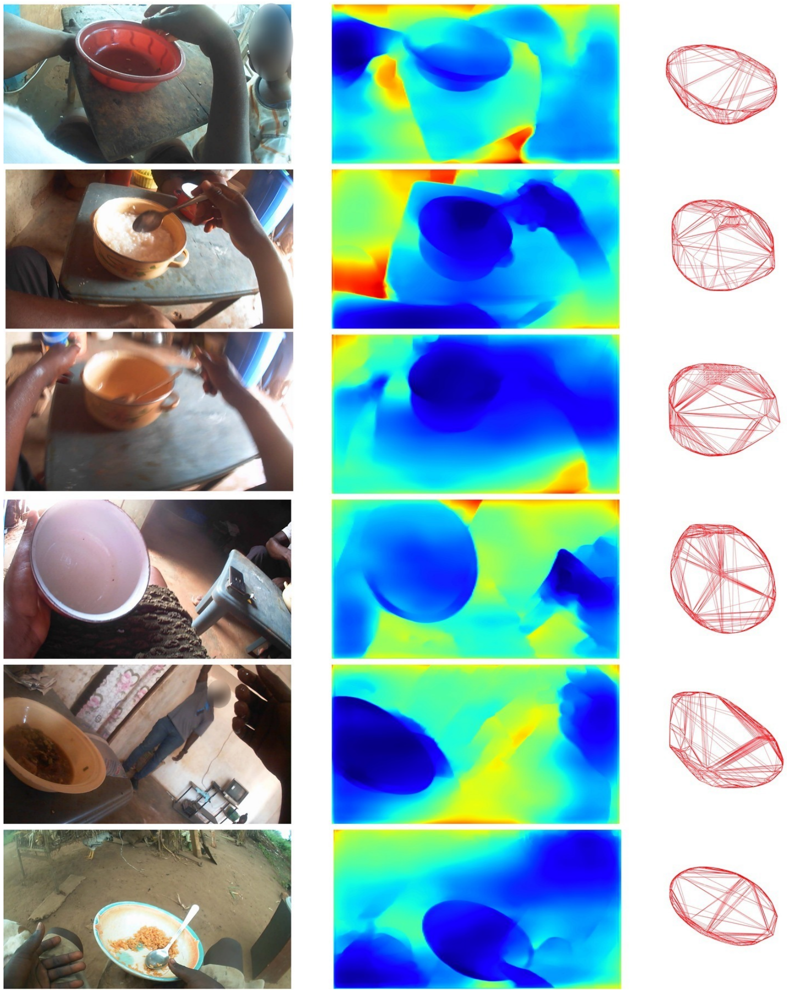

Supplementary Figure 4. 3D reconstruction of food containers using images captured under free-living conditions in the rural region of Ghana. Illustrative examples obtained through EgoDiet:3DNet are presented, detailing the 3D reconstruction of food containers for different eating scenarios. Currently, the process of selecting images to estimate the scale of food containers cannot yet be fully automated and requires some human involvement. Selecting images without hand obstructions and with empty containers enhances the performance of 3D reconstruction and improves the accuracy of scale determination. Additionally, including general objects of known or standard sizes, such as forks and spoons, in the images as references can aid in this process (i.e., a step to segment these objects needs to be added).

Supplementary Table 2. Absolute error in nutritional content across varied eating episodes in Study A

Episode #	Food Lists	Absolute Error of Consumed Portion Size (g) for each food item	Absolute Error of Protein (g)	Absolute Error of Fat (g)	Absolute Error of Carbohydrate (g)	Absolute Error of Energy (kcal)
1	Tilapia fish, onions, tomato soup, banku	53.0, 4.8, 14.7, 94.5	13.8	10.1	52.2	284.7
2	Yam, spinach stew, avocado	0.5, 12.9, 0.5	0.3	0.1	1.1	5.9
3	Tomato salad, rice dish	33.7, 98.1	4.0	3.2	33.3	177.5
4	Chapati, ugali, beef stew	91.4, 63.3, 128.0	1.4	0.3	59.1	229.2
5	Salted fish, yam, spinach stew	8.6, 96.2, 2.2	4.0	0.1	26.3	85.6
6	Banku, tomato soup, tilapia fish, onions	111.3, 79.7, 18.7, 2.7	4.6	2.4	62.3	204.6
7	Chicken, rice dish	10.4, 42.6	4.3	2.6	13.9	95.6
8	Chicken, rice dish	16.2, 32.4	2.9	0.8	10.5	23.1
9	Chicken, rice dish	17.5, 54.2	6.6	3.7	17.6	130.2
10	Salted fish, spinach stew, avocado, yam	1.7, 1.3, 0.9, 25.7	1.5	0.2	7.0	35.5

Note that this table quantifies the absolute error in nutrient estimation for various eating episodes within Study A. The absolute error for the consumed portion size is determined by the difference between the portion size ascertained by the EgoDiet system and the actual ground truth portion size of each eating episode. The absolute errors for carbohydrates, proteins, fats, and calories are computed subsequent to the conversion of the estimated portion sizes into nutrient content, utilizing nutritional database as a reference standard in this preliminary study. Note that passive monitoring can sometimes result in incomplete captures of the food (e.g., food items not centered in the image or partially obscured due to natural body movement). Therefore, even with the same types of food, there may be variations in errors. MAE of protein: 4.3g; MAE of fat: 2.3g; MAE of carbohydrate: 28.3g; MAE of energy: 127.2kcal.

Supplementary Table 3. Absolute error in nutritional content for each food item across varied eating episodes in Study A

Episode #	Food Lists	Absolute Error of Protein (g)	Absolute Error of Fat (g)	Absolute Error of Carbohydrate (g)	Absolute Error of Energy (kcal)
1	Tilapia fish, onions, tomato soup, banku	9.12, 0.03, 0.35, 5.03	7.16, 0.12, 0.19, 3.22	6.20, 0.31, 1.47, 47.8	125.62, 2.38, 8.69, 170.18
2	Yam, spinach stew, avocado	0.01, 0.35, 0.01	0, 0.13, 0.07	0.12, 1.25, 0.04	0.52, 7.25, 0.79
3	Tomato salad, rice dish	0.28, 3.77	0.10, 3.13	1.36, 31.9	6.74, 170.77
4	Chapati, ugali, beef stew	10.24, 1.14, 12.8	6.81, 1.14, 8.23	42.40, 24.05, 7.31	271.42, 112.66, 154.89
5	Salted fish, yam, spinach stew	5.38, 1.43, 0.06	0.2, 0.13, 0.02	0, 26.47, 0.21	24.84, 111.64, 1.25
6	Banku, tomato soup, tilapia fish, onions	5.92, 1.91, 3.21, 0.02	3.8, 1.03, 2.52, 0.07	56.29, 7.97, 2.18, 0.17	200.41, 47.04, 44.20, 1.35
7	Chicken, rice dish	2.66, 1.64	1.19, 1.36	0.01, 13.86	21.39, 74.21
8	Chicken, rice dish	4.14, 1.25	1.86, 1.03	0.02, 10.54	33.3, 56.42
9	Chicken, rice dish	4.47, 2.08	2.01, 1.73	0.02, 17.61	35.98, 94.26
10	Salted fish, spinach stew, avocado, yam	1.10, 0.03, 0.02, 0.38	0.04, 0.01, 0.13, 0.04	0, 0.12, 0.07, 7.07	5.06, 0.72, 1.39, 29.8

Supplementary Table 4. Absolute error in nutritional content across varied eating episodes under free-living conditions in Study B

Episode #	Food Lists	Absolute Error of Consumed Portion Size (g)	Absolute Error of Protein (g)	Absolute Error of Fat (g)	Absolute Error of Carbohydrate (g)	Absolute Error of Energy (kcal)
1	Rice dish	9.4	0.4	0.3	3.1	16.4
2	Rice dish	153.9	5.9	4.9	50.0	267.8
3	Spinach stew	82.7	2.2	0.8	8.0	46.3
4	Yam	71.8	1.1	0.1	19.8	83.3
5	Rice dish	245.2	9.4	7.8	79.7	426.7
6	Rice dish	31.5	1.2	1.0	10.3	54.9
7	Rice dish	225.9	8.7	7.2	73.4	393.1
8	Yam	37.3	0.6	0.1	10.3	43.3
9	Rice dish	227.4	8.7	7.3	73.9	395.7

Note that the food items in Study B were selected and prepared by the participants' families, which included various rice dishes. For the purposes of our study, all these dishes were categorized simply as 'rice dish'. Since our primary focus of this research is on portion size estimation rather than nutritional content analysis, our system categorizes them based on existing classes (i.e., our existing model focuses on broader food categories rather than fine-grained types), which may not accurately reflect the nutritional composition prepared by the participants. Nutritional analysis will be explored further in our future research. Additionally, it should be noted that the estimation error for portion size in Study B appears to be greater than in Study A. This is largely due to the fact that the food containers used in Study B have a relatively larger volume, allowing them to hold more food, particularly when participants are consuming rice dishes. This increases the absolute error in estimating the consumed portion size in gram. However, despite this, Egodiet can still achieve comparable performance in free-living conditions compared to 24-Hour Dietary Recall (24HR).

Supplementary Table 5. Mean absolute percentage error in estimating consumed portion size for each food category by the EgoDiet system and dietitians

	Chicken	Tomato salad	Chapati	Beef stew	Spinach stew	Rice dish	Yam
EgoDiet's estimation	45.0%	22.0%	60.1%	50.4%	4.5%	37.2%	26.6%
Dietitian's estimation	93.8%	52.3%	55.3%	24.0%	5.5%	13.0%	13.7%
	Avocado	Salted fish	Onions	Tilapia fish	Tomato soup	Banku	Ugali
EgoDiet's estimation	2.6%	54.3%	25.2%	14.3%	43.9%	37.1%	61.4%
Dietitian's estimation	15.6%	85.5%	43.1%	77.9%	64.2%	27.7%	12.6%

Note that the table presents the mean absolute percentage error (MAPE) in predicting consumed portion sizes for various food categories by the EgoDiet system and dietitians. The average MAPE for EgoDiet's estimation is 34.6%, while the average MAPE for dietitians' estimation is 41.7%.

Supplementary References

1. Qiu, J. *et al.* Counting bites and recognizing consumed food from videos for passive dietary monitoring. *IEEE JBHI* (2020).

2. Bahador, N., Ferreira, D., Tamminen, S., Kortelainen, J. *et al.* Deep learning–based multimodal data fusion: Case study in food intake episodes detection using wearable sensors. *JMIR mHealth uHealth* **9**, e21926 (2021).

3. Doulah, A. *et al.* Energy intake estimation using a novel wearable sensor and food images in a laboratory (pseudo-free-living) meal setting: quantification and contribution of sources of error. *Int. J. Obes.* **46**, 2050–2057 (2022).

4. Elbassuoni, S. *et al.* Deepnova: A deep learning nova classifier for food images. *IEEE Access* **10**, 128523–128535 (2022).

5. Elbassuoni, S. *et al.* Capturing children food exposure using wearable cameras and deep learning. *PLOS Digit. Heal.* **2**, e0000211 (2023).

6. Fontana, J. M., Farooq, M. & Sazonov, E. Detection and characterization of food intake by wearable sensors. In *Wearable Sensors*, 541–574 (Elsevier, 2021).

7. Vu, T., Lin, F., Alshurafa, N. & Xu, W. Wearable food intake monitoring technologies: A comprehensive review. *Computers* **6**, 4 (2017).
